# Di­chlorido­[2-(pyridin-2-yl-κ*N*)-1,5-naphthyridine-κ*N*^1^]zinc(II)

**DOI:** 10.1107/S2414314625007643

**Published:** 2025-09-05

**Authors:** Kodai Tsubokawa, Takuro Iida, Kiyoshi Tsuge, Hideki Ohtsu

**Affiliations:** a Graduate School of Science and Engineering University of Toyama, 3190 Gofuku Toyama 930-8555 Japan; Goethe-Universität Frankfurt, Germany

**Keywords:** crystal structure, zinc(II) complex, NAD^+^/NADH model ligand

## Abstract

The title zinc(II) complex, [ZnCl_2_(C_13_H_9_N_3_)], crystallizes in the triclinic space group *P*1. The coordination environment around the zinc(II) ion in the title complex can be described as a distorted tetra­hedron formed by the two N atoms of the NAD^+^/NADH model ligand pn [pn = 2-(pyridin-2-yl)[1,5]naphthyridine] and two Cl^−^ ions. There are *π*–*π* stacking inter­actions in the crystal packing of the title compound.

## Abstract 2

The title compound, [ZnCl_2_(C_13_H_9_N_3_)], is an example for new zinc(II) complexes with the NAD^+^/NADH model ligand pn [pn = 2-(pyridin-2-yl)[1,5]naphthyridine]. The central zinc(II) atom exhibits a slightly distorted tetra­hedral coordination geometry (*τ*_4_ = 0.88) with a bidentate pn ligand and two monodentate Cl^−^ ions. In the crystal, two mol­ecules of the title compound show aromatic *π*–*π* inter­actions.

## Graphical abstract

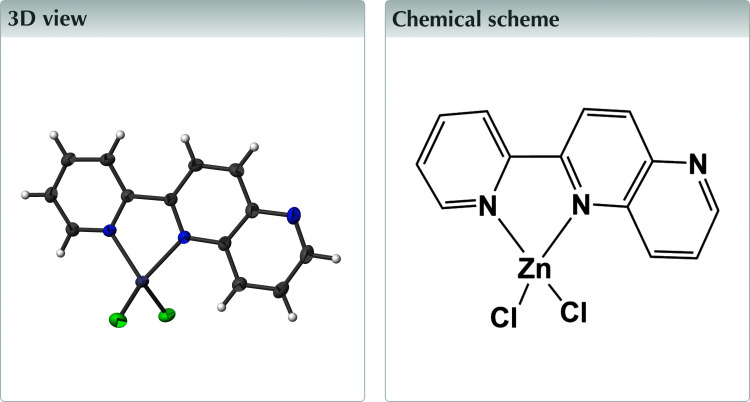

## Structure description

Photo-driven carbon dioxide (CO_2_) reduction has been one of the most attractive approaches to address global energy and environmental problems because of its capacity to transform CO_2_ into value-added chemical compounds, such as formic acid and carbon monoxide, under mild conditions by utilizing solar energy (Wang *et al.*, 2022 ▸). Transition-metal mol­ecular catalysts are an important tool and play a central role in the roadmap to achieve efficient and novel CO_2_ photoreduction into valuable chemicals (Kumagai *et al.*, 2022 ▸). Our motivation for investigating transition-metal complexes with a coenzyme NAD^+^/NADH model ligand is based on their potential as candidates for photocatalytic CO_2_ reduction. The synthesis and use of the NAD^+^/NADH model ligand pbn [pbn = 2-(pyridin-2-yl)benzo[*b*][1,5]naphthyridine] was first reported by Koizumi & Tanaka (2005 ▸), and we have previously developed a novel photocatalytic CO_2_ reduction process to produce formic acid using a Ru-based pbn complex (Ohtsu & Tanaka, 2012 ▸; Ohtsu *et al.*, 2015 ▸, 2019 ▸).

In order to further develop transition-metal NAD^+^/NADH model complexes, substituent tuning of NAD^+^/NADH model ligands offers a potentially powerful means not only to control catalytic activity of the complexes but also to confer new reactivity on the complexes. However, the synthetic pathway to introduce substituents into the benzonaphthyridine skeleton of the pbn ligand is considerably difficult.

As part of our ongoing investigation of transition-metal complexes bearing various substituted NAD^+^/NADH model ligands, we have focused on the non-substituted NAD^+^/NADH model ligand pn [pn = 2-(pyridin-2-yl)[1,5]naphthyridine] synthesized by Singh & Thummel (2009 ▸), which can possess the potential to facilitate the introduction of substituents through a straightforward synthetic process. A new zinc(II) complex with a pn ligand has been structurally characterized and is reported in this paper.

**Figure 1:**
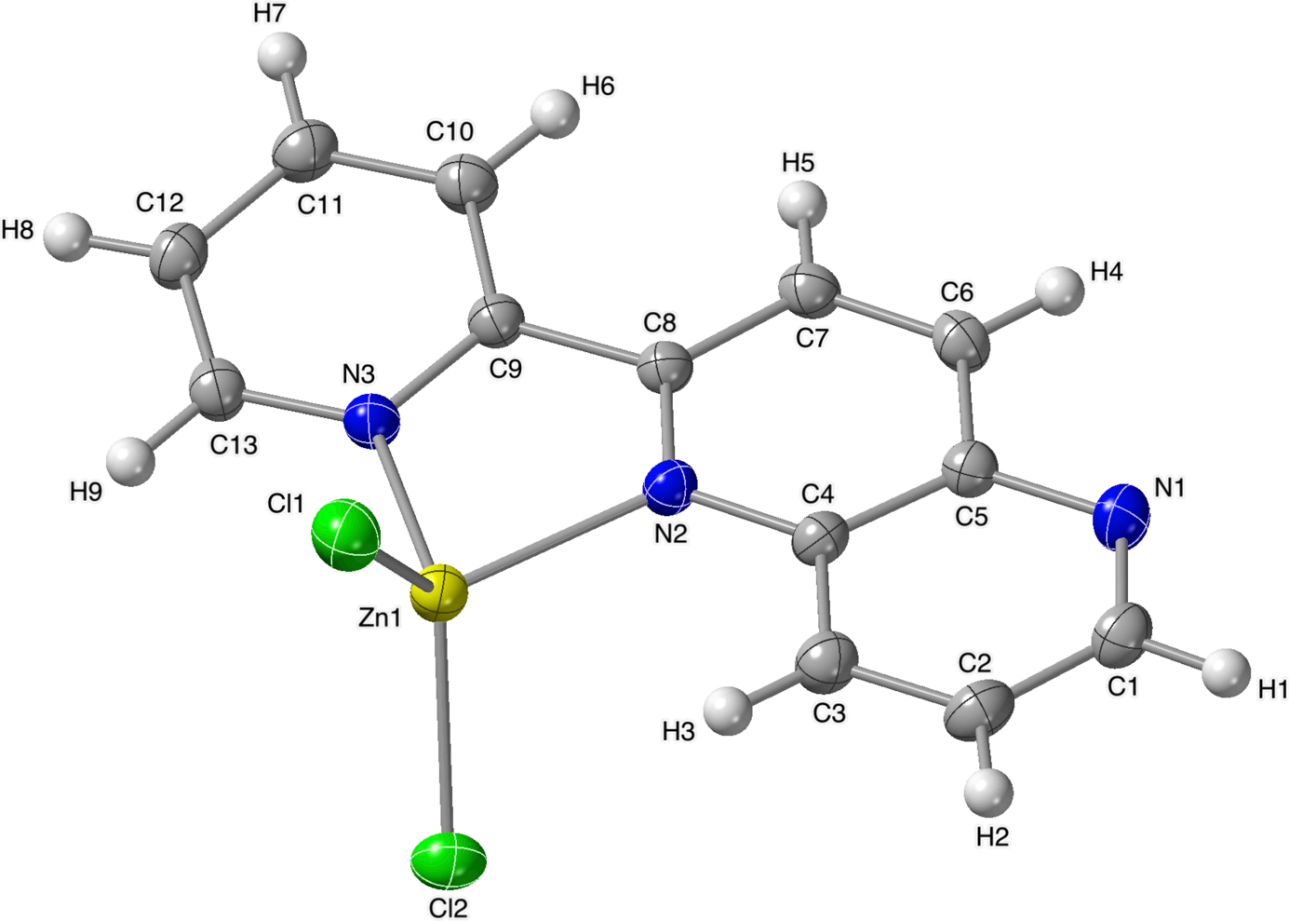
The mol­ecular structure of the title compound with displacement ellipsoids for non-hydrogen atoms at the 50% probability level.

The mol­ecular structure of the title complex, [ZnCl_2_(pn)], is shown in Fig. 1 ▸ and selected geometrical data are listed in Table 1 ▸. The zinc(II) ion in [ZnCl_2_(pn)] has a tetra­coordinate structure formed by the two N atoms of pn ligand [Zn1—N2 = 2.0909 (14) Å, Zn1—N3 = 2.0560 (14) Å] and two Cl^−^ ions [Zn1—Cl1 = 2.2137 (6) Å, Zn1—Cl2 = 2.2161 (6) Å]. The qu­anti­tative difference in four-coordinate geometry is indicated by an index of *τ*_4_. The value can range from *τ*_4_ = 1 for a perfect tetra­hedral geometry to *τ*_4_ = 0 for a perfect square planar geometry (Yang *et al.*, 2007 ▸). The *τ*_4_ value for the zinc(II) ion of the title complex is obtained as *τ*_4_ = 0.88 by using the equation *τ*_4_ = [360–(*α*+*β*)]/141 (Yang *et al.*, 2007 ▸), where *α* = N3—Zn1—Cl2 [120.94 (4)°], *β* = Cl1—Zn1—Cl2 [114.81 (2)°]. Thus, the coordination environment of the zinc(II) ion in [ZnCl_2_(pn)] is a slightly distorted tetra­hedron. The pyridine ring and the naphthyridine ring system in the pn ligand are twisted to give a dihedral angle of 10.57 (5)° between the two least-squares planes.

**Figure 2:**
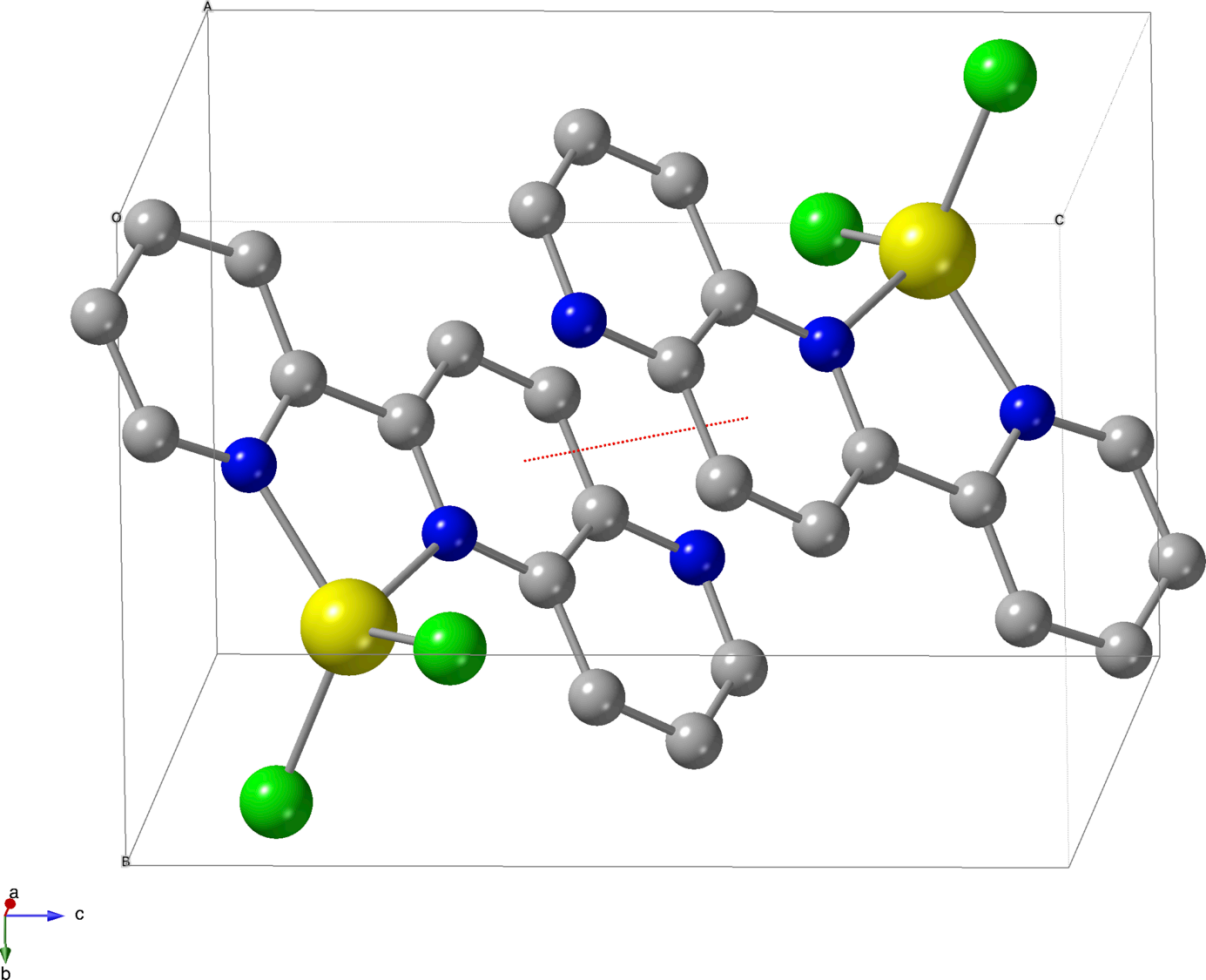
Part of the crystal structure showing a *π*–*π* inter­action (red dotted line). Zn atoms are represented in yellow, Cl in green, N in blue, and C in gray. Hydrogen atoms are omitted for clarity.

The crystal packing of the title complex is shown in Fig. 2 ▸. There are noteworthy *π*–*π* stacking inter­actions between neighboring naphthyridine ring systems of the pn ligand, with a centroid–centroid distance of 3.625 (1) Å. No other significant or inter­esting inter­molecular inter­actions are observed.

## Synthesis and crystallization

The NAD^+^/NADH model ligand, 2-(pyridin-2-yl)[1,5]naphthyridine abbreviated as pn, was prepared according to the literature procedure (Singh & Thummel, 2009 ▸).

To a di­chloro­methane solution (4.0 ml) of pn (36.44 mg, 17.6 mmol) was added dropwise ZnCl_2_ (23.95 mg, 17.6 mmol) in aceto­nitrile (4.0 ml), and the resulting solution was left to stand for a few days at room temperature. Light-yellow crystals of the title compound [ZnCl_2_(pn)] were obtained (yield; 44.48 mg, 73.7%). Elemental analysis, found: C 45.32, H 2.69, N 12.18%; calculated for C_13_H_9_Cl_2_N_3_Zn: C 45.45, H 2.64, N 12.23%.

## Refinement

Crystal data, data collection and structure refinement details are summarized in Table 2 ▸.

## Supplementary Material

Crystal structure: contains datablock(s) global, I. DOI: 10.1107/S2414314625007643/bt4180sup1.cif

Structure factors: contains datablock(s) I. DOI: 10.1107/S2414314625007643/bt4180Isup2.hkl

CCDC reference: 2480689

Additional supporting information:  crystallographic information; 3D view; checkCIF report

## Figures and Tables

**Table 1:** Selected geometric parameters (Å, °)

Zn1—N3	2.0560 (14)	Zn1—Cl1	2.2137 (6)
Zn1—N2	2.0909 (14)	Zn1—Cl2	2.2161 (6)
			
N3—Zn1—N2	79.59 (6)	N3—Zn1—Cl2	120.94 (4)
N3—Zn1—Cl1	113.63 (4)	N2—Zn1—Cl2	108.77 (4)
N2—Zn1—Cl1	113.74 (4)	Cl1—Zn1—Cl2	114.81 (2)

**Table 2:** Experimental details

Crystal data	
Chemical formula	[ZnCl_2_(C_13_H_9_N_3_)]
*M*_r_	343.52
Crystal system, space group	Triclinic, *P* 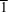
Temperature (K)	173
*a*, *b*, *c* (Å)	8.0634 (15), 8.6146 (17), 10.2087 (19)
α, β, γ (°)	86.492 (6), 78.622 (6), 70.336 (5)
*V* (Å^3^)	654.6 (2)
*Z*	2
Radiation type	Mo *K*α
μ (mm^−1^)	2.27
Crystal size (mm)	0.23 × 0.11 × 0.07

Data collection	
Diffractometer	Rigaku R-AXIS RAPID
Absorption correction	Multi-scan (*ABSCOR*; Rigaku, 1995 ▸)
*T*_min_, *T*_max_	0.630, 0.853
No. of measured, independent and observed [*F*^2^ > 2.0σ(*F*^2^)] reflections	6486, 2992, 2693
*R*_int_	0.034
(sin θ/λ)_max_ (Å^−1^)	0.649

Refinement	
*R*[*F*^2^ > 2σ(*F*^2^)], *wR*(*F*^2^), *S*	0.026, 0.069, 1.03
No. of reflections	2992
No. of parameters	172
H-atom treatment	H-atom parameters constrained
Δρ_max_, Δρ_min_ (e Å^−3^)	0.43, −0.28

Computer programs: *RAPID-AUTO* (Rigaku, 2001 ▸), *SHELXT2018/2* (Sheldrick, 2015*a* ▸), *SHELXL2019/3* (Sheldrick, 2015*b* ▸), *CrystalStructure* (Rigaku, 2019 ▸), *CrystalMaker* (Palmer, 2014 ▸) and *publCIF* (Westrip, 2010 ▸).

## full crystallographic data

### Dichlorido[2-(pyridin-2-yl-κN)-1,5-naphthyridine-κN1]zinc(II) Crystal data

**Table:**

[ZnCl_2_(C_13_H_9_N_3_)]	*Z* = 2
*M**_r_* = 343.52	*F*(000) = 344.00
Triclinic, *P*1	*D*_x_ = 1.743 Mg m^−^^3^
*a* = 8.0634 (15) Å	Mo *K*α radiation, λ = 0.71075 Å
*b* = 8.6146 (17) Å	Cell parameters from 4464 reflections
*c* = 10.2087 (19) Å	θ = 2.0–27.5°
α = 86.492 (6)°	µ = 2.27 mm^−^^1^
β = 78.622 (6)°	*T* = 173 K
γ = 70.336 (5)°	Block, colorless
*V* = 654.6 (2) Å^3^	0.23 × 0.11 × 0.07 mm

### Dichlorido[2-(pyridin-2-yl-κN)-1,5-naphthyridine-κN1]zinc(II) Data collection

**Table:**

Rigaku R-AXIS RAPID diffractometer	2693 reflections with *F*^2^ > 2.0σ(*F*^2^)
Detector resolution: 10.000 pixels mm^-1^	*R*_int_ = 0.034
ω scans	θ_max_ = 27.5°, θ_min_ = 2.5°
Absorption correction: multi-scan (ABSCOR; Rigaku, 1995)	*h* = −10→10
*T*_min_ = 0.630, *T*_max_ = 0.853	*k* = −11→11
6486 measured reflections	*l* = −13→12
2992 independent reflections	

### Dichlorido[2-(pyridin-2-yl-κN)-1,5-naphthyridine-κN1]zinc(II) Refinement

**Table:**

Refinement on *F*^2^	Secondary atom site location: difference Fourier map
*R*[*F*^2^ > 2σ(*F*^2^)] = 0.026	Hydrogen site location: inferred from neighbouring sites
*wR*(*F*^2^) = 0.069	H-atom parameters constrained
*S* = 1.03	*w* = 1/[σ^2^(*F*_o_^2^) + (0.0387*P*)^2^ + 0.1345*P*] where *P* = (*F*_o_^2^ + 2*F*_c_^2^)/3
2992 reflections	(Δ/σ)_max_ = 0.001
172 parameters	Δρ_max_ = 0.43 e Å^−^^3^
0 restraints	Δρ_min_ = −0.28 e Å^−^^3^
Primary atom site location: structure-invariant direct methods	

### Dichlorido[2-(pyridin-2-yl-κN)-1,5-naphthyridine-κN1]zinc(II) Special details

**Table:**

Geometry. All esds (except the esd in the dihedral angle between two l.s. planes) are estimated using the full covariance matrix. The cell esds are taken into account individually in the estimation of esds in distances, angles and torsion angles; correlations between esds in cell parameters are only used when they are defined by crystal symmetry. An approximate (isotropic) treatment of cell esds is used for estimating esds involving l.s. planes.
Refinement. Refinement was performed using all reflections. The weighted R-factor (wR) and goodness of fit (S) are based on F^2^. R-factor (gt) are based on F. The threshold expression of F^2^ > 2.0 sigma(F^2^) is used only for calculating R-factor (gt).H atoms were located in a difference map and refined as riding on their parent atoms with C–H = 0.95 Å and with *U*_iso_(H) = 1.2 *U*_eq_(C).

### Dichlorido[2-(pyridin-2-yl-κN)-1,5-naphthyridine-κN1]zinc(II) Fractional atomic coordinates and isotropic or equivalent isotropic displacement parameters (Å^2^)

**Table:**

	*x*	*y*	*z*	*U*_iso_*/*U*_eq_	
Zn1	0.52702 (3)	0.80210 (2)	0.18747 (2)	0.02563 (8)	
Cl1	0.70577 (6)	0.89381 (6)	0.27656 (5)	0.03363 (12)	
Cl2	0.29182 (6)	0.99685 (6)	0.13125 (5)	0.03711 (12)	
N1	0.1233 (2)	0.5604 (2)	0.59856 (15)	0.0327 (3)	
N2	0.43844 (18)	0.62744 (18)	0.30470 (13)	0.0220 (3)	
N3	0.66057 (19)	0.59482 (18)	0.07077 (14)	0.0235 (3)	
C1	0.0609 (2)	0.7111 (3)	0.64747 (18)	0.0326 (4)	
H1	−0.029364	0.732608	0.725885	0.039*	
C2	0.1173 (2)	0.8429 (2)	0.59282 (18)	0.0305 (4)	
H2	0.068503	0.948033	0.635055	0.037*	
C3	0.2437 (2)	0.8172 (2)	0.47777 (18)	0.0284 (4)	
H3	0.284141	0.903886	0.438031	0.034*	
C4	0.3122 (2)	0.6578 (2)	0.41992 (16)	0.0227 (3)	
C5	0.2495 (2)	0.5333 (2)	0.48381 (16)	0.0250 (3)	
C6	0.3234 (2)	0.3738 (2)	0.42718 (18)	0.0287 (4)	
H6	0.284985	0.286671	0.468330	0.034*	
C7	0.4511 (2)	0.3448 (2)	0.31252 (17)	0.0259 (3)	
H7	0.501827	0.237854	0.273490	0.031*	
C8	0.5059 (2)	0.4762 (2)	0.25336 (15)	0.0215 (3)	
C9	0.6415 (2)	0.4540 (2)	0.12667 (15)	0.0218 (3)	
C10	0.7425 (2)	0.3016 (2)	0.06984 (18)	0.0280 (4)	
H10	0.728308	0.203757	0.111003	0.034*	
C11	0.8650 (2)	0.2936 (2)	−0.04844 (18)	0.0303 (4)	
H11	0.937152	0.189843	−0.088050	0.036*	
C12	0.8812 (2)	0.4371 (2)	−0.10788 (17)	0.0291 (4)	
H12	0.961660	0.434432	−0.190011	0.035*	
C13	0.7767 (2)	0.5854 (2)	−0.04455 (17)	0.0280 (4)	
H13	0.788006	0.684666	−0.084677	0.034*	

### Dichlorido[2-(pyridin-2-yl-κN)-1,5-naphthyridine-κN1]zinc(II) Atomic displacement parameters (Å^2^)

**Table:**

	*U*^11^	*U*^22^	*U*^33^	*U*^12^	*U*^13^	*U*^23^
Zn1	0.02615 (12)	0.01684 (12)	0.03053 (12)	−0.00643 (9)	0.00227 (9)	−0.00346 (8)
Cl1	0.0383 (2)	0.0267 (2)	0.0385 (2)	−0.01520 (19)	−0.0046 (2)	−0.00282 (18)
Cl2	0.0341 (2)	0.0217 (2)	0.0473 (3)	−0.00115 (18)	−0.0029 (2)	−0.00052 (19)
N1	0.0306 (8)	0.0319 (9)	0.0285 (7)	−0.0073 (7)	0.0053 (7)	0.0006 (6)
N2	0.0207 (6)	0.0200 (7)	0.0236 (6)	−0.0062 (5)	−0.0003 (6)	−0.0030 (5)
N3	0.0251 (7)	0.0181 (7)	0.0247 (6)	−0.0057 (6)	−0.0007 (6)	−0.0010 (5)
C1	0.0269 (9)	0.0360 (11)	0.0263 (8)	−0.0041 (8)	0.0039 (7)	−0.0017 (7)
C2	0.0252 (8)	0.0295 (10)	0.0309 (9)	−0.0023 (7)	−0.0014 (7)	−0.0085 (7)
C3	0.0275 (8)	0.0243 (9)	0.0308 (9)	−0.0075 (7)	−0.0003 (8)	−0.0046 (7)
C4	0.0188 (7)	0.0239 (9)	0.0234 (8)	−0.0049 (6)	−0.0030 (7)	−0.0015 (6)
C5	0.0230 (8)	0.0258 (9)	0.0232 (7)	−0.0058 (7)	−0.0023 (7)	0.0019 (6)
C6	0.0322 (9)	0.0226 (9)	0.0301 (8)	−0.0106 (7)	−0.0015 (8)	0.0038 (7)
C7	0.0292 (8)	0.0187 (8)	0.0268 (8)	−0.0052 (7)	−0.0032 (7)	−0.0004 (6)
C8	0.0211 (7)	0.0198 (8)	0.0221 (7)	−0.0053 (6)	−0.0035 (7)	−0.0001 (6)
C9	0.0220 (7)	0.0205 (8)	0.0219 (7)	−0.0060 (6)	−0.0031 (7)	−0.0011 (6)
C10	0.0322 (9)	0.0195 (9)	0.0295 (8)	−0.0071 (7)	−0.0010 (8)	−0.0019 (7)
C11	0.0299 (9)	0.0236 (9)	0.0318 (9)	−0.0049 (7)	0.0023 (8)	−0.0073 (7)
C12	0.0272 (8)	0.0311 (10)	0.0250 (8)	−0.0081 (7)	0.0024 (7)	−0.0034 (7)
C13	0.0298 (9)	0.0247 (9)	0.0271 (8)	−0.0094 (7)	0.0005 (7)	0.0013 (7)

### Dichlorido[2-(pyridin-2-yl-κN)-1,5-naphthyridine-κN1]zinc(II) Geometric parameters (Å, º)

**Table:**

Zn1—N3	2.0560 (14)	C4—C5	1.408 (2)
Zn1—N2	2.0909 (14)	C5—C6	1.410 (3)
Zn1—Cl1	2.2137 (6)	C6—C7	1.371 (2)
Zn1—Cl2	2.2161 (6)	C6—H6	0.9500
N1—C1	1.314 (3)	C7—C8	1.410 (2)
N1—C5	1.366 (2)	C7—H7	0.9500
N2—C8	1.328 (2)	C8—C9	1.496 (2)
N2—C4	1.369 (2)	C9—C10	1.382 (2)
N3—C13	1.340 (2)	C10—C11	1.390 (2)
N3—C9	1.352 (2)	C10—H10	0.9500
C1—C2	1.406 (3)	C11—C12	1.378 (3)
C1—H1	0.9500	C11—H11	0.9500
C2—C3	1.369 (3)	C12—C13	1.386 (3)
C2—H2	0.9500	C12—H12	0.9500
C3—C4	1.414 (2)	C13—H13	0.9500
C3—H3	0.9500		
			
N3—Zn1—N2	79.59 (6)	N1—C5—C6	119.26 (16)
N3—Zn1—Cl1	113.63 (4)	C4—C5—C6	117.99 (15)
N2—Zn1—Cl1	113.74 (4)	C7—C6—C5	119.77 (16)
N3—Zn1—Cl2	120.94 (4)	C7—C6—H6	120.1
N2—Zn1—Cl2	108.77 (4)	C5—C6—H6	120.1
Cl1—Zn1—Cl2	114.81 (2)	C6—C7—C8	119.04 (16)
C1—N1—C5	116.46 (16)	C6—C7—H7	120.5
C8—N2—C4	119.22 (14)	C8—C7—H7	120.5
C8—N2—Zn1	114.20 (11)	N2—C8—C7	122.39 (15)
C4—N2—Zn1	126.40 (11)	N2—C8—C9	115.80 (14)
C13—N3—C9	118.69 (15)	C7—C8—C9	121.80 (15)
C13—N3—Zn1	126.24 (12)	N3—C9—C10	121.59 (15)
C9—N3—Zn1	114.60 (11)	N3—C9—C8	115.16 (14)
N1—C1—C2	125.12 (17)	C10—C9—C8	123.25 (15)
N1—C1—H1	117.4	C9—C10—C11	119.02 (16)
C2—C1—H1	117.4	C9—C10—H10	120.5
C3—C2—C1	118.96 (17)	C11—C10—H10	120.5
C3—C2—H2	120.5	C12—C11—C10	119.61 (17)
C1—C2—H2	120.5	C12—C11—H11	120.2
C2—C3—C4	117.95 (17)	C10—C11—H11	120.2
C2—C3—H3	121.0	C11—C12—C13	118.17 (16)
C4—C3—H3	121.0	C11—C12—H12	120.9
N2—C4—C5	121.59 (15)	C13—C12—H12	120.9
N2—C4—C3	119.67 (16)	N3—C13—C12	122.88 (17)
C5—C4—C3	118.74 (15)	N3—C13—H13	118.6
N1—C5—C4	122.75 (16)	C12—C13—H13	118.6
			
C5—N1—C1—C2	−1.7 (3)	C4—N2—C8—C9	179.39 (14)
N1—C1—C2—C3	1.8 (3)	Zn1—N2—C8—C9	3.94 (17)
C1—C2—C3—C4	−0.4 (3)	C6—C7—C8—N2	0.0 (3)
C8—N2—C4—C5	−1.3 (2)	C6—C7—C8—C9	−178.65 (15)
Zn1—N2—C4—C5	173.50 (12)	C13—N3—C9—C10	2.0 (2)
C8—N2—C4—C3	177.99 (15)	Zn1—N3—C9—C10	−170.68 (13)
Zn1—N2—C4—C3	−7.2 (2)	C13—N3—C9—C8	−178.52 (15)
C2—C3—C4—N2	179.83 (15)	Zn1—N3—C9—C8	8.83 (18)
C2—C3—C4—C5	−0.8 (2)	N2—C8—C9—N3	−8.6 (2)
C1—N1—C5—C4	0.3 (3)	C7—C8—C9—N3	170.21 (15)
C1—N1—C5—C6	179.19 (17)	N2—C8—C9—C10	170.93 (15)
N2—C4—C5—N1	−179.78 (16)	C7—C8—C9—C10	−10.3 (2)
C3—C4—C5—N1	0.9 (2)	N3—C9—C10—C11	−0.7 (3)
N2—C4—C5—C6	1.4 (2)	C8—C9—C10—C11	179.82 (16)
C3—C4—C5—C6	−177.98 (16)	C9—C10—C11—C12	−1.2 (3)
N1—C5—C6—C7	−179.57 (16)	C10—C11—C12—C13	1.8 (3)
C4—C5—C6—C7	−0.7 (3)	C9—N3—C13—C12	−1.4 (3)
C5—C6—C7—C8	0.0 (3)	Zn1—N3—C13—C12	170.35 (13)
C4—N2—C8—C7	0.6 (2)	C11—C12—C13—N3	−0.5 (3)
Zn1—N2—C8—C7	−174.83 (13)		
